# *Discs large 5*, an Essential Gene in *Drosophila*, Regulates Egg Chamber Organization

**DOI:** 10.1534/g3.115.017558

**Published:** 2015-03-19

**Authors:** Eve Reilly, Neha Changela, Tatyana Naryshkina, Girish Deshpande, Ruth Steward

**Affiliations:** * Department of Molecular Biology, Rutgers University, Waksman Institute, Cancer Institute of New Jersey, Piscataway, New Jersey 08854; † Department of Molecular Biology, Princeton University, Princeton, New Jersey

**Keywords:** *Drosophila*, oogenesis, stalk and polar cells, embryonic germ cells, E-cadherin

## Abstract

Discs large 5 (Dlg5) is a member of the MAGUK family of proteins that typically serve as molecular scaffolds and mediate signaling complex formation and localization. In vertebrates, Dlg5 has been shown to be responsible for polarization of neural progenitors and to associate with Rab11-positive vesicles in epithelial cells. In *Drosophila*, however, the function of Dlg5 is not well-documented. We have identified *dlg5* as an essential gene that shows embryonic lethality. *dlg5* embryos display partial loss of primordial germ cells (PGCs) during gonad coalescence between stages 12 and 15 of embryogenesis. Loss of Dlg5 in germline and somatic stem cells in the ovary results in the depletion of both cell lineages. Reduced expression of Dlg5 in the follicle cells of the ovary leads to a number of distinct phenotypes, including defects in egg chamber budding, stalk cell overgrowth, and ectopic polar cell induction. Interestingly, loss of Dlg5 in follicle cells results in abnormal distribution of a critical component of cell adhesion, E-cadherin, shown to be essential for proper organization of egg chambers.

Discs large 5 (Dlg5) is a member of the membrane-associated guanylate kinase (MAGUK) family of proteins that typically serve as molecular scaffolds and mediate signaling complex formation and localization. MAGUK family proteins are highly conserved and carry a core PDZ-SH3-GUK domain. They are divided into 10 subfamilies, including the *discs large* subfamily, named for homology to *Drosophila discs large* (Lewander *et al.* 1990). PDZ (PSD-95, Discs Large, ZO 1) domains are composed of approximately 100 amino acid residues and are often localized to the plasma membrane (Oliva *et al.* 2012). Many PDZ domains are involved in assembly of signaling complexes in signal transduction pathways and cell polarity determination, and they have been implicated in tumorigenesis (Oliva *et al.* 2012; Subbaiah *et al.* 2011).

First identified in humans, *dlg5* was originally named *P-dlg* due to its expression in the placenta and the prostate gland and for its homology to *Drosophila discs large (dlg1)*. Dlg1 is the founding member of the Discs Large family, first studied because of its overgrowth phenotype; mutant larvae fail to metamorphose and die with highly overgrown discs (Woods and Bryant 1989). *Dlg1* is a key regulator of apico-basal polarity in neuroblasts and other polarized processes such as cell invasion (Roberts *et al.* 2012). Similar to *dlg1*, *dlg5* has been demonstrated to function during determination and maintenance of epithelial cell polarity, during cell adhesion, migration, and proliferation, and during signaling in vertebrates (Liu *et al.* 2014).

Polymorphisms in Dlg5 interfere with its scaffolding function and are associated with inflammatory bowel disease in humans (Stoll *et al.* 2004). Despite this association, little is known about Dlg5. *Drosophila* Dlg5 and human Dlg5 (Discs large 5 homolog) share the same general domain architecture, with several conserved protein-binding domains. Alignment of the amino acid sequences of homologous regions of *Drosophila* and human Dlg5 shows that they share 45–54% sequence homology (Roberts *et al.* 2012). Thus, *dlg5* may be functionally conserved in flies and mammals, as is characteristic of many of the other MAGUK proteins.

In vertebrates, Dlg5 is responsible for polarization of neural progenitors and associates with Rab11-positive vesicles in epithelial cells to serve as a scaffold for polarized delivery of cadherin-catenin containing complexes to the plasma membrane (Nechiporuk *et al.* 2007) (Chang *et al.* 2010). In *Drosophila*, Dlg5 maintains cluster cohesion among migrating border cells in the ovary (Aranjuez *et al.* 2012). How Dlg5 exerts this function, however, remains largely unknown. Given the importance of the polarizing influence of Dlg5 in other species (Liu *et al.* 2014), we decided to further investigate the role of *dlg5* in *Drosophila* oogenesis.

Oogenesis in *Drosophila* begins when a germline stem cell (GSC), located at the tip of the germarium (Figure 1A), undergoes an asymmetric division, forming a cystoblast and a GSC. After four divisions with incomplete cytokinesis, the cystoblast ultimately forms a 16-cell cyst. While moving through the germarium, the dividing cystoblast is surrounded by somatic escort cells. At the transition from germarium region 2a to 2b, these escort cells are exchanged with a monolayer of follicle cells, resulting in the formation of a discrete egg chamber (Figure 1A) (Morrison and Spradling 2008). Egg chamber formation requires three crucial steps: first, follicle cell proliferation as a result of asymmetric division of follicle stem cells (FSCs); next, follicle cell migration and encapsulation of the cyst; and, finally, differentiation of stalk and polar cells to separate the newly formed egg chamber from its antecedent (Dobens and Raftery 2000). Stalk cells and polar cells cease dividing in the germarium soon after cell-fate specification, unlike follicle cells that proliferate until approximately stage 7 (Tworoger *et al.* 1999; Margolis and Spradling 1995; Zhang and Kalderon 2000). Egg chambers continuously "bud off" from the germarium and move posteriorly as they continue to develop.

**Figure 1:**
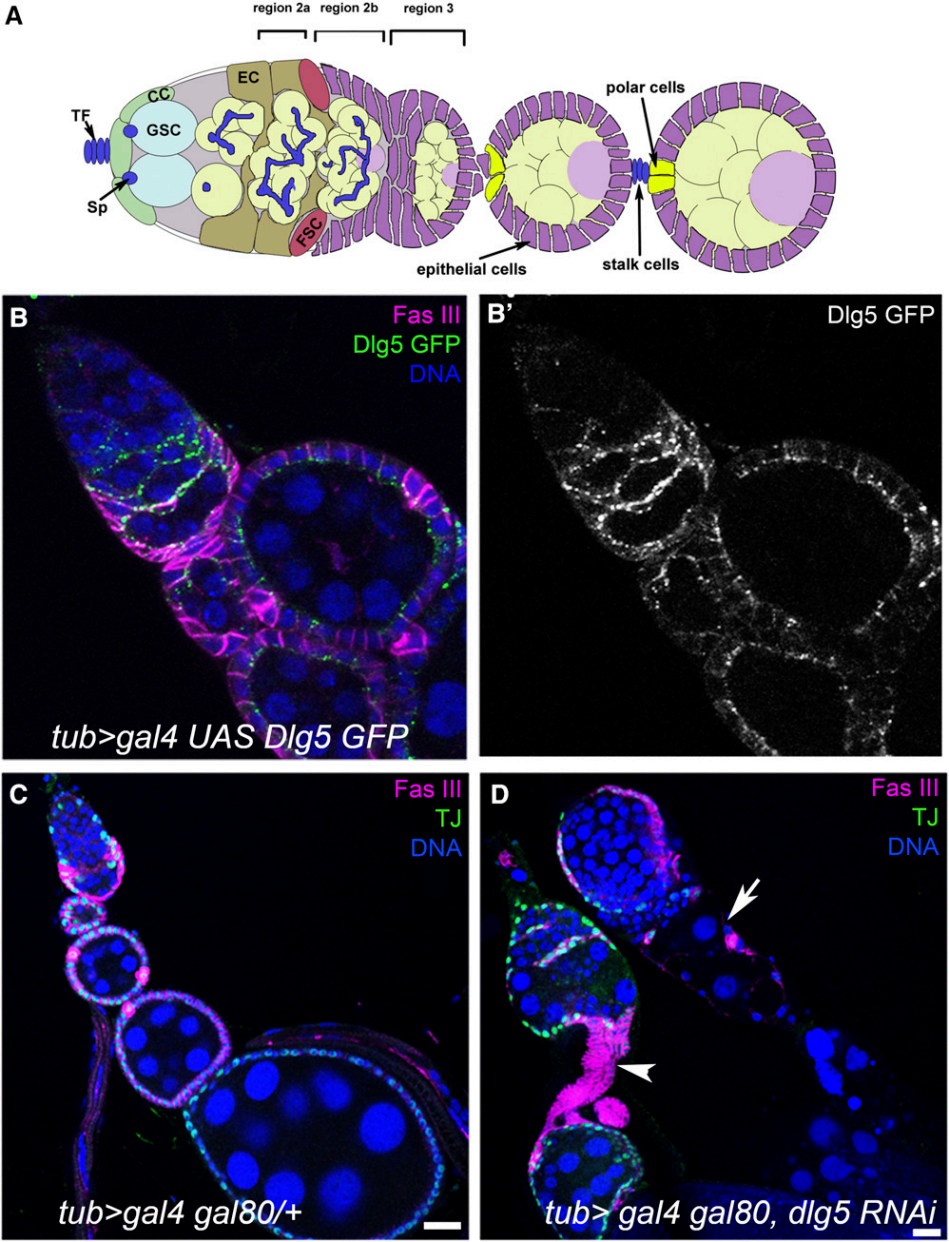
Dlg5 in ovaries. (A) Drawing of *Drosophila* ovariole that contains a germarium and three egg chambers. GSC, germline stem cells; FSC, follicle stem cells; CC, cap cells; EC, escort cells; Sp, spectrosome; TF, terminal filament. (B, B′) Ovariole expressing Dlg5::GFP under the control of the *tub*-Gal4 driver. Note the punctate apical localization of Dlg5 in follicle cells. FasIII marks follicle cells. (C) Control ovariole, driver only. (D) Ovariole with reduced levels of Dlg5 in follicle cells (Dlg5 KD). Note the long, multicellular stalks (arrowhead) and degenerate egg chambers (arrow). Scale bar, 20 μm.

Here, we show that *dlg5* is essential in embryos, and by clonal analysis we find that *dlg5* is required in FSCs and GSCs, because persistent *dlg5* germline and follicle cell clones were not detected. Knockdown (KD) of *dlg5* in FSCs and their daughters resulted in egg chambers that never developed beyond stage 6. Phenotypes include ectopic polar cell clusters, stalk cell overgrowth, and the formation of disorganized egg chambers, sometimes containing two 16-cell cysts. We further show that the distribution of E-cadherin in Dlg5 KD follicle cells is affected. The phenotype associated with depletion of Dlg5 in somatic cells of the ovary ultimately agrees with the phenotype observed in *dlg5* null clones. In both genetic backgrounds, mutant clones and cells depleted of Dlg5 are eventually lost. Finally, *dlg5* embryos display germ cell loss specifically during mid to late stages of embryogenesis, suggesting a requirement of *dlg5* during germ cell migration and gonad coalescence.

## Materials and Methods

### Fly strains and generation of a *dlg5* null allele

The fly stock expressing GFP-tagged Dlg5 (w*; *P{w[+mC]=UAS-CG6509.GFP}3)*, deposited by H. Richardson, was obtained from the Bloomington Stock Center, as was the *dlg5* RNAi line primarily used in these experiments (*UAS-CG6509^GD11943^*RNAi) and the *CyO* balancer expressing beta-galactosidase under the *engrailed* (*en*) promoter, *CyO en-LacZ* (*CyO*, *P{en1}wg^en11^*). Additional *dlg5* RNAi lines (*P{GD11943}v22496* and *P{KK104086}VIE-260B*) were obtained from VDRC. The KD efficiency of *P{GD11943}v22496* was assessed previously (Aranjuez *et al.* 2012). Because KD of *UAS-CG6509^GD11943^* and *P{GD11943}v22496* resulted in comparable phenotypes, we concluded that both lines affect *dlg5* expression to similar degrees. The *dlg5^D48^* allele was generated by imprecise excision of a *P{SUPor-P}CG6509^KG00748^* (*dlg5^KG00748^*, Bloomington Stock Center) transposable element. To excise the *P*-element, the insertion stock was crossed with a stock expressing transposase (McGregor *et al.* 2002; Robertson *et al.* 1988). Flies carrying potential excision chromosomes were crossed to *w^118^*; *Df(2L)BSC242/CyO*, a deficiency stock uncovering the *dlg5* gene region (Bloomington Stock Center). Putative *dlg5* mutants were identified by lethality.

To determine the lethal period of the excision alleles ,they were crossed to a *CyO* balancer carrying a transgene that expresses GFP under the *ubiquitous* (*ubi*) promoter (*CyO*, *P{Ubi-GFP.S65T}PAD1*, obtained from the Bloomington stock center). These flies were crossed with *w^118^*; *Df(2L)BSC242/ CyO*, *P{Ubi-GFP.S65T}*PAD1 and set-up for egg-laying on agarose plates. The plates were changed after 8 hr and inspected for survival of nongreen first instar larvae after 24 hr. At least 200 green balancer larvae were counted for each cross.

The *c587-Gal4;UAS-CD8*::*GFP* driver was obtained from T. Schüpbach, Princeton University; when GFP expression is not shown in the Figures, we refer to this driver as *c587-Gal4*. All other drivers were obtained from the Bloomington Stock Center.

### Clonal analysis

Wild-type negatively marked clones were induced and analyzed as described byFox *et al.* (2008). Newly eclosed adult *y w P[hs70FLP];ubi-GFP.nls FRT 40A/FRT 40A dlg5^D48^* flies were exposed twice within 24 hr to 38° for 1 hr per exposure. Mutant clones were induced in *y w P[hs70FLP];ubi-GFP.nls FRT 40A/FRT 40A dlg5^D48^* flies. Persistent clones were induced and inspected at least three times, but the clones were counted in only one experiment. *Ubi-GFP.nls FRT40A* flies were obtained from the Bloomington Stock Center and *y w P[hs70FLP]* flies were obtained from K. McKim, Rutgers University.

### Immunostaining

Dissection, fixing, and staining of *Drosophila* ovaries were performed as described previously (Minakhina *et al.* 2014). Primary antibodies and concentration used, respectively, are as follows: guinea pig anti-Traffic Jam (1:5000; a gift from D. Godt, University of Toronto), mouse anti-FasIII [1:20; Developmental Studies Hybridoma Bank (DSHB)], rat anti-Vasa (1:20), rat anti-DEcad (E-cadherin, 1:150; DSHB), mouse anti-Eyes Absent (1:100; DSHB), and mouse anti-3A9 (alpha spectrin, 1:20; DSHB). Phalloidin 488 (Invitrogen) and secondary antibodies (Jackson Laboratories) were used at concentrations of 1:300. Hoechst 33258 (1:5000) was used to stain DNA. Images were captured using a Leica DM IRBE SP2 or Leica TSC SP5 laser scanning confocal microscope (40× oil objective), analyzed with Leica Microsystems Software, and further processed using Adobe Photoshop.

To observe germ cell migration, 0- to 16-hr-old embryos from the *dlg5*/*CyO engrailed-lacz* stock were collected and fixed using 4% Paraformaldehyde/Heptane mix. Embryos were stained with rabbit anti-Vasa (1:1000; gift from Paul Lasko, McGill University) and rabbit anti-B-galactosidase antibodies (purchased from Cappel; 1:10,000). Subsequently, horseradish peroxidase–coupled anti-Rabbit secondary antibody was used and the signal was visualized using standard histochemical procedures. Vasa antibody labels the primordial germ cells. The homozygous mutant embryos were identified by the absence of beta-galactosidase signal (Deshpande *et al.* 2009).

### Mapping the *dlg5^D48^* deletion

PCR analysis was used to determine the breakpoints of the deletion generated in the *P*-element excision mutant *dlg5^D48^*. Because of the perdurance of maternally contributed GFP, DNA from single dechorionated embryos was used for this analysis. PCR of DNA isolated from wild-type and mutant embryos was done in parallel (Mathew *et al.* 2009). The *P*-element excision mutant stock was maintained over a GFP expressing *CyO* balancer (*CyO*, *P{Ubi-GFP.S65T}PAD1*); homozygous mutant embryos were identified by selecting for lack of GFP at 4-6 hr after egg-lay, as soon as GFP-negative embryos could be identified.

#### Primer sequences:

Primer 6: 5′-AAA AGG TTG TTA GTG CGT GCA-3′
Primer 11: 5′- GTG TGA CCG CGG AGT GAC-3′
Primer 5: 5′- TCACACTGGTGACGTTTTA-3′
Primer 4: 5′- CGCTCCCGGTAGTAGTTG-3′
Primer 10: 5′-CTG CAG GCG CAG TAC AAG TC-3′
Primer 3: 5′-TGA TGG TGC GGA TGG TAG-3′
Primer 27: 5′- AGT ATG TGG CAT TAA CAT GCG GG-3′
Primer 28: 5′- GAG TCG CCA GGC TCG TCT TTA-3′
Primer 32: 5′-GAC TTC TCC AAC CTC TTC-3′
Primer 1: 5′- GTT GCT TAG CTA GTC GCG-3′
Primer 23: 5′- TCC AGA ACG CCT ATG CTT TCG-3′
Primer 24: 5′- CGG AAG AGT GGT ATC GCC CA-3′

## Results

### Dlg5 is localized in follicle cells

Dlg proteins have been shown to localize asymmetrically within tissues (Woods *et al.* 1996). To investigate Dlg5 protein distribution in *Drosophila* ovarian tissue in the absence of a specific anti-Dlg5 antibody, we expressed GFP-tagged Dlg5 *(w^+^*; *P{w[+mC]=UAS-CG6509.GFP}3)* under the control of the *tubulin (tub)-Gal4* driver, active in somatic cells of the ovary (Figure 1, B and B′). In escort and follicle cells, the Dlg5::GFP protein is seen in puncta. In escort cells, Dlg5::GFP accumulated on the extensions that protrude between the germline cysts in regions 2a and 2b (Figure 1, A and B′). In follicle cells, the protein is mostly found on the apical side, facing the germ cells. Thus, similar to vertebrates, polarized distribution of Dlg5 is conserved in flies and hence may be functionally relevant.

### Isolation of a *dlg5* null allele by imprecise *P*-element excision

To determine the effect of loss of Dlg5 on oogenesis, we set out to generate a *dlg5* null mutant. We mobilized the *P{SUPor-P}* insertion, marked with *yellow^+^* (*y^+^*) and *white^+^* (*w^+^*) in *dlg5 ^KG00748^*. This allele is homozygous lethal at late third instar larval stage, and also lethal in late third instar larvae over *Df(2L)BSC24*, a chromosomal deletion that uncovers *dlg5*. We obtained a total of 59 y *w* excision chromosomes, of which 47 were viable over *Df(2L)BSC242*, indicating that the lethality observed on the original chromosome was due to the *P*-element insertion. Twelve lines were confirmed to be lethal over *Df(2L)BSC242*. Homozygotes and hemizygotes (over *Df(2L)BSC242*) of all 12 putative imprecise *P*-element excision mutant lines were embryonic lethal (see *Materials and Methods*). Although *dlg5^KG00748^* mutants are lethal during larval stages, *dlg5^D48^* homozygous and hemizygous animals die as embryos. Consequently, no larvae hatch, indicating that *dlg5* has an essential function in embryos, while *dlg5^KG00748^* appears to represent a partial loss of function allele.

One of the lethal excision mutations, *dlg5^D48^*, was randomly selected for further analysis. We collected single *dlg5^D48^* embryos to map the breakpoints of the deletion created by the *P*-element excision by PCR (Supporting Information, Figure S1). Although we were able to detect PCR fragments derived from DNA upstream of the *P*-element insertion site in both wild-type and mutant embryos, we were unable to amplify fragments downstream of it (primers 10 and 3; see *Materials and Methods*). Similarly, we were unable to amplify the region flanking the *P*-element insertion site in *dlg5^D48^* (primers 5 and 4). However, DNA sequences distal to primer 3 could be amplified in both wild-type and mutant DNA. The genes flanking *dlg5*, therefore, appear to be unaffected. We hypothesize that part of the 11-kb *P*-element may have undergone a rearrangement but that *P*-element-derived sequences remain in the gene. We conclude that the sequences abutting the *P*-element insertion site on the distal side are missing and that the distal deletion breakpoint lies between primer 10 and primer 27. Together, these results indicate that the N-terminal end of the protein is missing. Based on these observations it is likely that *dlg5^D48^* represents a null allele.

### *Dlg5* is required in the ovarian germline and soma

Because *dlg5^D48^* is embryonic lethal, we used clonal analysis via FRT-mediated site-specific mitotic recombination (see *Materials and Methods*) (Xu and Rubin 1993) to determine the requirement of *dlg5* in the germline and soma. Two types of clones, persistent and transient, are observed in ovaries and are classified based on the time elapsed between clone induction and clonal analysis. Most clones observed 2 to 5 d ACI (after clone induction) are in the follicle cells of maturing egg chambers. These transient clones originate from a recombination event of a nonstem cell and are lost at the end of oogenesis when the follicle cells disintegrate (Margolis and Spradling 1995). Persistent clones result from a recombination event in a stem cell and will typically last throughout the life of the fly as the stem cell continuously self-renews with each division.

**Figure 2:**
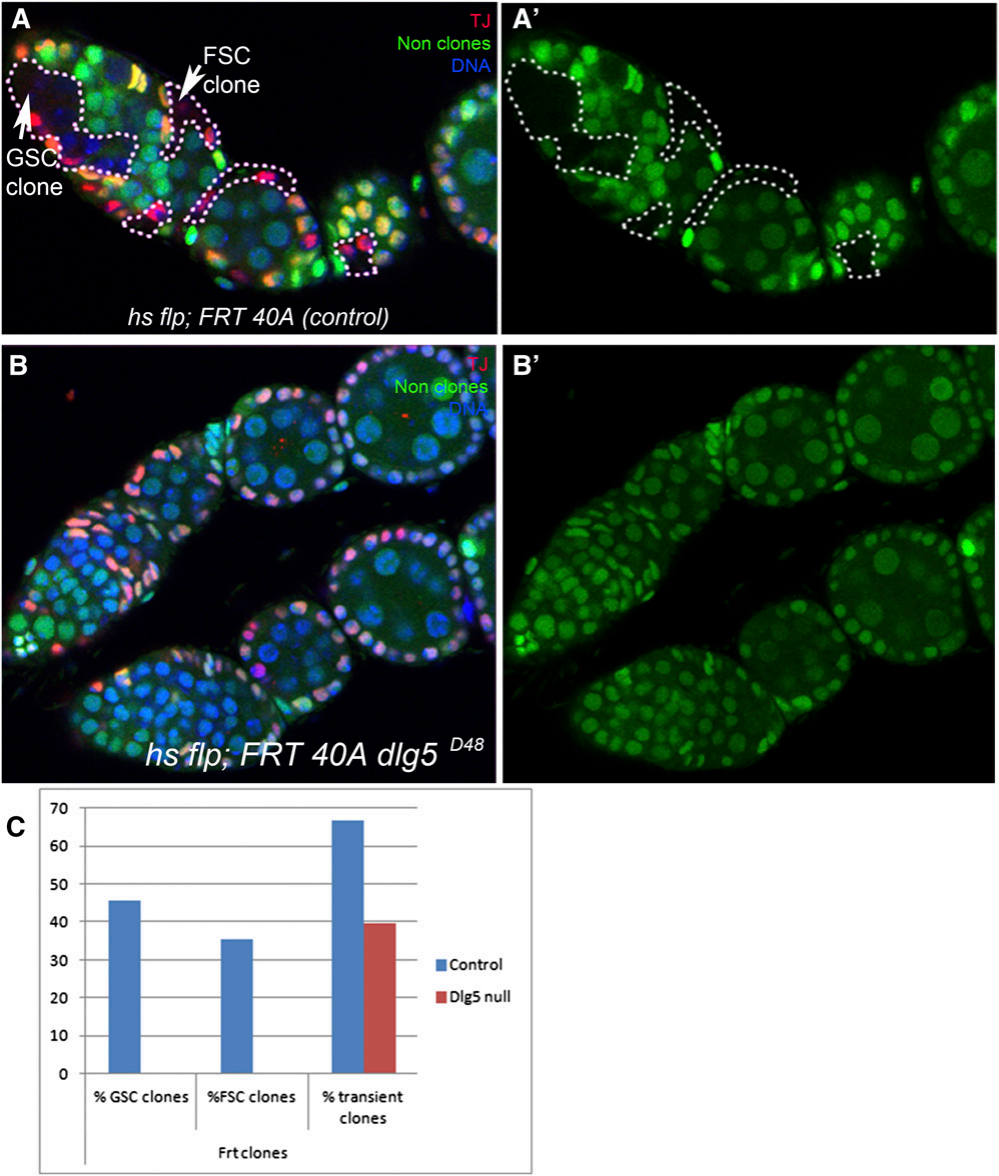
Dlg5 requirement in germ line and follicle stem cells. (A, A′) Control ovariole dissected 10 d after clone induction (ACI), showing germline and somatic persistent stem cell clones. (B, B′) No germline or somatic *dlg5^D48^* clones were detected in ovarioles 10 d ACI. (C) Graph noting the number of ovarioles containing wild-type and *dlg5^D48^* persistent and transient clones obtained by the original FRT technique (Xu and Rubin 1993). Clones in (A, A′) are marked by the absence of GFP and somatic cells by the presence of Traffic Jam protein (red). DNA, blue.

Clones were induced in newly eclosed females and analyzed 10 d ACI (see *Materials and Methods*). In control flies, 48 ovarioles were counted. Of these, 22 had persistent stem cell-derived germ line clones and 17 had persistent clones derived from follicle stem cells (46% and 35%, respectively) (Figure 2C). Thirty-six ovarioles from *ywP[hs70FLP]*; *Ubi-GFP nls.2L FRT 40A/FRT 40A dlg5^D48^* flies were investigated for the presence of clones. No persistent *dlg5* clones were observed in the germline or follicle cells (Figure 2), indicating that *dlg5* is essential for the normal division and possibly maintenance of both germline and follicle stem cells. The percentage of ovarioles with transient wild type and *dlg5* clones were comparable for both time points. After 5 d ACI, 64% of wild-type ovarioles (∼150 ovarioles) contained transient clones, whereas *dlg5* mutant clones were only detected in 40% of ovarioles (∼150 ovarioles) (Figure 2C). Transient *dlg5* clones were smaller than control clones (not shown), supporting the idea that loss of *dlg5* affects cell division or cell viability. From these results we can conclude that Dlg5 is essential in both germline and somatic stem cells, and it is likely required in all follicle cells throughout oogenesis.

### Reduced levels of Dlg5 in follicle cells leads to egg chamber defects

Flies in which Dlg5 levels have been ubiquitously depleted by expression of *dlg5* RNAi under a *tubulin (tub)-gal4* ubiquitous driver die at the third instar larval stage with ∼2% of animals surviving into the pupal stage. To circumvent the lethality associated with general loss of Dlg5, RNAi expression was inhibited until after eclosion using the *Gal4-Gal80* system (Suster *et al.* 2004). Animals were reared at 18° and newly eclosed flies were held for 4 d at 29° to induce RNAi expression before ovaries were dissected and immunostained for analysis. To address the possibility of off-target effects, the phenotype of two different RNAi lines targeting *dlg5* were compared (see *Materials and Methods*). Both produced the same phenotypes, and so it is unlikely that the observed defects are a consequence of off-target effects.

Depletion of Dlg5 in the somatic cells of the ovary using the general *tub* driver resulted in severe morphological defects. One striking phenotype was the overgrown stalk-like structures (Figure 1D, arrowhead). Ovarioles containing degenerating follicles with only nurse cell nuclei and surrounding follicle cells remaining were also observed (Figure 1D, arrow).

**Figure 3:**
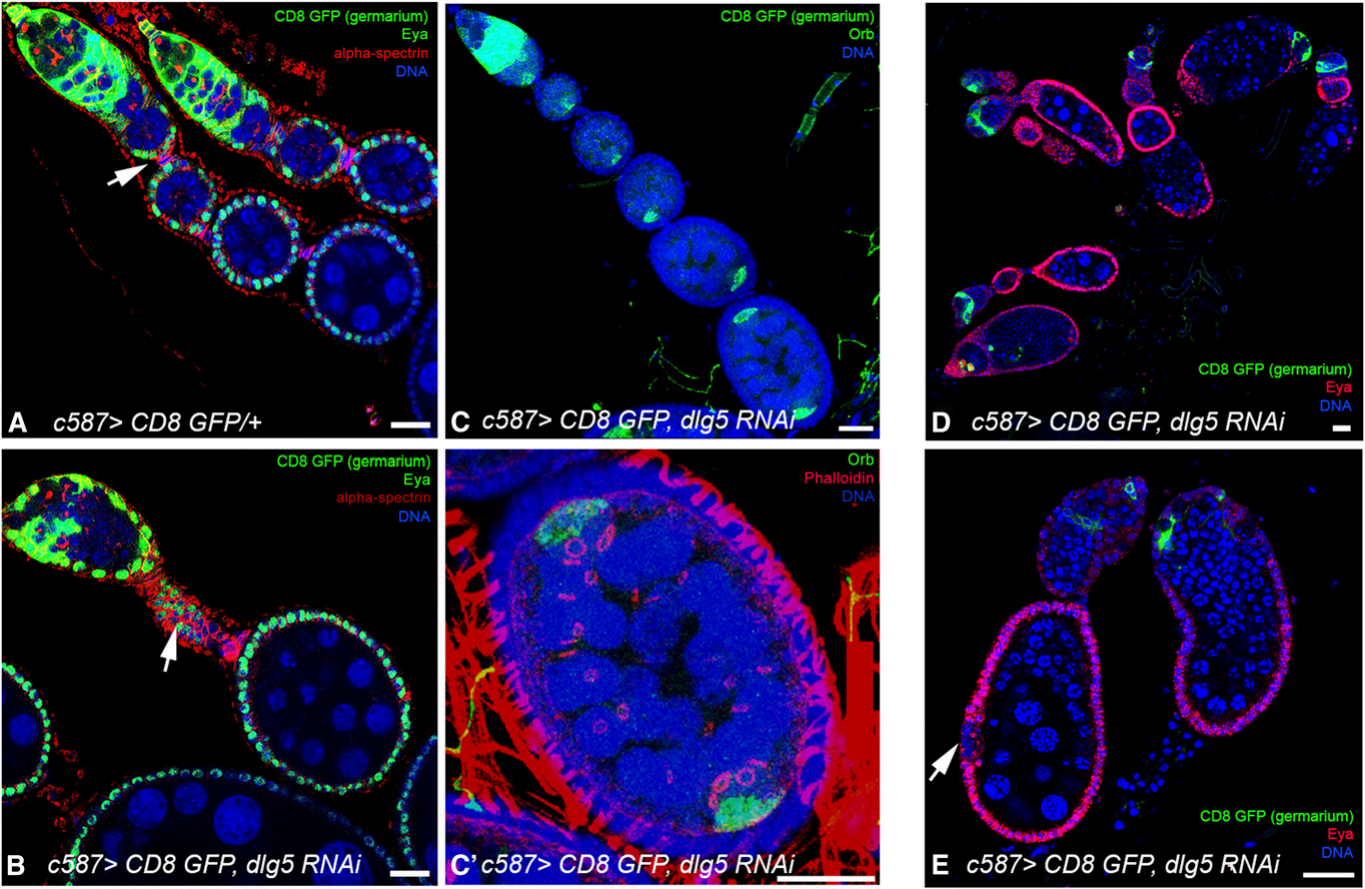
Phenotypes resulting from depletion of Dlg5 in follicle cells. (A) Control ovarioles, driver only. (B) Dlg5 KD ovariole 4 d after RNAi induction. Note the abnormal stalk between two Dlg5 KD egg chambers (arrows). (C) Dlg5 KD results in disorganized egg chambers with abnormal numbers of oocytes. (C′) Enlarged picture of egg chamber with two oocytes. Both oocytes are connected by four ring canals with the neighboring nurse cells, indicating the egg chambers contain two 16-cell cysts. (D) Ovarioles 10 d after RNAi induction. Most germaria and egg chambers are disorganized and no mature egg chambers are detected. (E) Higher magnification of ovarioles 10 d after RNAi induction. The packaging of the germline cells is abnormal. Note the patch of Eyes Absent (Eya)-negative follicle cells. (A, B, C, D, and E) Activity of the *c587* driver (c587 > CD8::GFP) in the germarium is shown in green. (A, B) alpha-spectrin is shown in red, Eya is shown in green. (C′) Actin (phalloidin) is shown in red. (C, C′) Orb (oocyte) is shown in green. (D and E) Eya protein shown in red. In all panels: DNA, blue; scale bar, 20 μm.

Given the apical localization of Dlg5 in follicle cells, we depleted the protein using the *c587-Gal4* driver, which is most highly expressed in somatic cells of the ovary, to drive expression of *dlg5* RNAi (Figure 3A) (Song *et al.* 2004). Females were transferred to 29° on eclosion to induce RNAi expression and ovaries were dissected 4 and 10 d later. Experiments were repeated at least twice and a minimum of 300 wild-type and KD ovarioles were inspected. In 4-d-old females, 10% of ovarioles showed normal morphology, whereas the other 90% contained abnormal germaria and egg chambers. The most striking phenotype was the overgrown stalk-like structures observed in 84% of ovarioles (Figure 3B, arrow). Some mutant egg chambers were separated by elongated stalks made up of more than the usual four to six cells, whereas others were divided by massively overgrown stalks that had failed to intercalate properly (Figure 3B). In these abnormal stalks, instead of the typical single column of cells, each stalk had at least two columns of cells. Although the organization of the stalks is aberrant, the cells themselves look similar to wild-type cells and have retained basal contact with their neighboring cell.

Fifteen percent of ovarioles contained at least one egg chamber with two oocytes (Figure 3, C and C′). To confirm that the increased nurse cell and oocyte number within enlarged egg chambers was not the product of a defect in germline cell divisions, the number of ring canals in each cell were counted. Each oocyte never contained more than the expected four ring canals formed by four successive divisions (Figure 3, C and C′), clearly indicating that defective packaging of germline cysts by follicle cells underlies the "multiple cyst" phenotype. The inclusion of multiple cysts within an egg chamber often results from abnormal polar and stalk cell specification, as well as delayed differentiation of these cell populations (Grammont and Irvine 2001; Lopez-Schier and St. Johnston 2001). The observed stalk cell and polar cell phenotypes suggest a defect at the level of cyst cell encapsulation.

In 10-d-old females, the ovaries had strikingly enhanced phenotypes (Figure 3, D and E). At most ∼1% of ovarioles showed normal morphology and ∼50% of ovarioles contained either an abnormal-looking germarium in an otherwise empty sac of somatic sheath cells or a germarium with a few discrete egg chambers. In some cases, follicles appear not to have separated from the germarium, and a number of germline cysts appeared to be degenerating. Abnormal compound cysts were found in the other 50%.

Together these results indicate the primary requirement of *dlg5* in follicle cells for the proper organization of the egg chamber as well as stalk formation. The results suggest that abnormal patterning ultimately results in the loss of follicle cells. They further underline the importance of the follicle epithelium to the integrity of the egg chamber and survival of germ line cells.

### Loss of Dlg5 in follicle cells leads to ectopic polar cells

**Figure 4:**
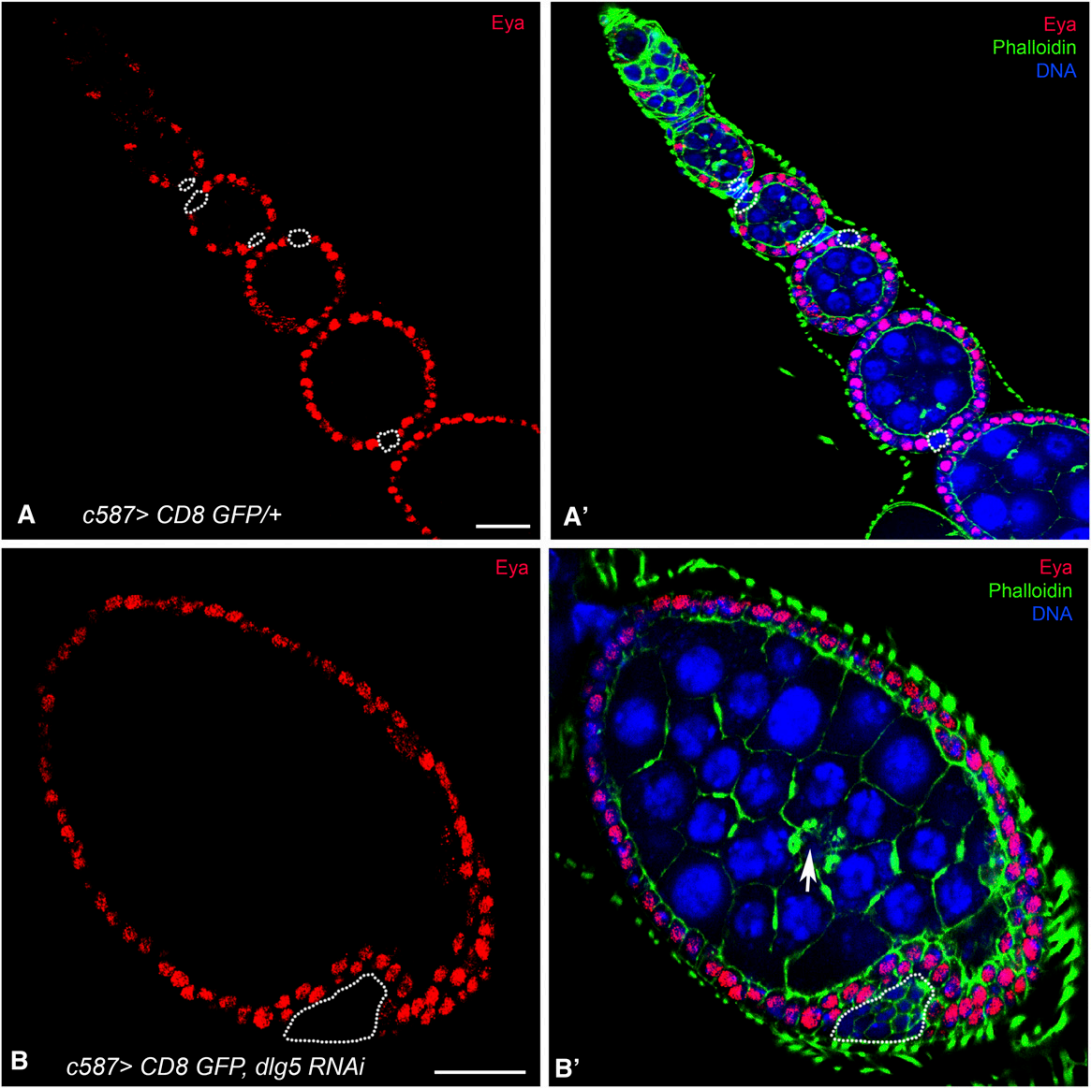
Polar cell numbers are increased in Dlg5 KD egg chambers. (A, A′) Control ovariole, driver only. Polar cells, marked by the absence of Eyes Absent (Eya) staining (red), are highlighted. (B, B′) Dlg5 KD egg chamber. Note the increase of Eya-negative polar cells and the oocyte (arrow) in the center of the egg chamber surrounded by four ring canals (actin, green). DNA, blue; scale bar, 20 μm.

Fasciclin III (Fas III) is highly expressed in immature follicle cells until egg chamber stages 2–3, when the level in most follicle cells is reduced except in polar cells, which stand out due to their strong FasIII staining (Figure 1A, B, and C). In addition to the observed stalk cell overgrowth, many Dlg5 KD egg chambers displayed ectopic FasIII expression (Figure 1D). Closer examination showed clusters of FasIII-positive cells on the surface of the follicular epithelium surrounding the egg chambers. The location and characteristic shape of these cells suggested that they were polar cell clusters. This was supported by further immunostaining, which revealed that these cells were negative for Eya (Figure 4B), indicating that they had fully differentiated into polar cells. Eyes Absent (Eya) is a repressor of polar cell fate and its absence is sufficient to induce a polar cell fate (Figure 4) (Bai and Montell 2002; Chang *et al.* 2013). Typically, polar cells appear at the anterior and posterior termini of each egg chamber (Figure 4A); however, in mutant egg chambers large patches of up to 25 polar cells often appear at random on the surface of the egg chamber (Figure 4B; Figure 3E, arrowhead). This phenotype was observed in at least 5% of *c587-Gal4-dlg5* RNAi ovarioles at d 4, and in >10% of egg chambers at 10 d.

### Dlg5 is required for germ cell migration

Because of the requirement of *dlg5* in migrating border cells in the ovary (Aranjuez *et al.* 2012) and the embryonic lethal phenotype associated with *dlg5*, we sought to investigate whether the gene is also essential during germ cell migration and gonad morphogenesis in embryos (for a detailed review see Kunwar *et al.* 2006). The primitive embryonic gonad in *Drosophila* consists of the somatic gonadal precursor cells (SGPs) and the primordial germ cells (PGCs). These two cell types are initially located in different regions of the embryo. The SGPs are mesodermal in origin and are specified by zygotically active genes during mid-embryogenesis in parasegments 10–13. By contrast, the PGCs are formed by precocious cellularization at the posterior pole of embryos at the syncytial blastoderm stage and are specified by maternal determinants.

The PGCs follow a stereotypical trajectory to ultimately coalesce with the SGPs into a gonad in a temporally coordinated manner. This migratory journey begins at gastrulation, when the PGCs are carried into the interior of the embryo by the mid-gut invagination. The PGCs in each group migrate laterally to come into contact with the gonadal mesoderm on either side of the embryo. PGCs align themselves in a row with the SGPs in parasegments 10–13, and these juxtaposed cells coalesce into the embryonic gonad. A combination of repulsive and attractive cues guides PGC migration through the mid-gut and toward the SGPs (Van Doren *et al.* 1998; Santos and Lehmann 2004; Deshpande and Schedl 2005; Kunwar *et al.* 2006).

**Figure 5:**
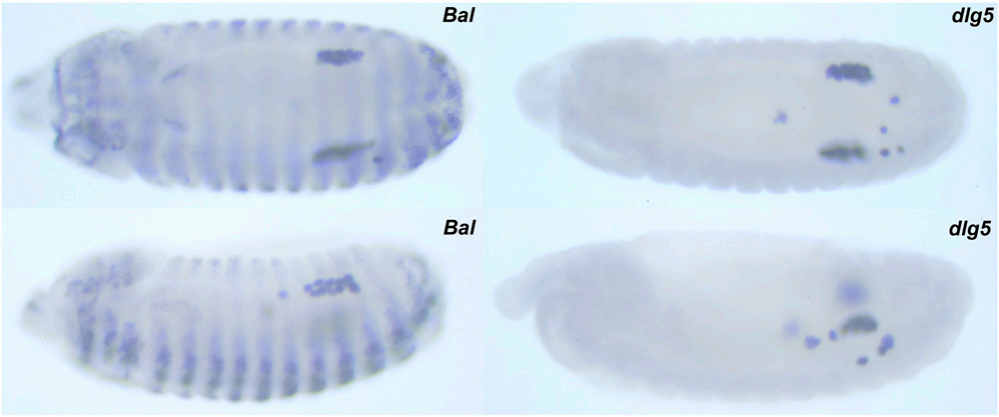
*Dlg5* embryos show defects in gonad formation. *Dlg5^D48^* embryos show variable germ cell migration defects and germ cell loss. While properly coalesced gonads are visible in the mutant embryos, several germ cells are scattered away from the coalesced gonads. *Dlg5^D48^*/*CyO en-lacZ*, control embryos recognized by the striped beta galactosidase staining. All embryos are approximately stage 13.

Because *dlg5^D48^* embryos are lethal, we analyzed embryos derived from *dlg5^D48^*/*CyO en-LacZ* flies to assess the requirement of *dlg5* during germ cell migration and gonad morphogenesis (Figure 5). Embryos were stained with anti-Vasa and anti-B-galactosidase antibodies. Although Vasa antibody labeled migrating germ cells, the B-galactosidase-specific staining allowed us to distinguish between the homozygous mutant embryos from the balancer embryos, which served as controls (Figure 5). In *dlg5^D48^* embryos, PGC specification and earlier phases of germ cell migration were relatively normal. Near wild-type numbers of PGCs were internalized within the gut pocket and also successfully traversed through the mid-gut epithelial wall. By stage 12 of embryogenesis, however, germ cell migration defects became apparent, as in a subset of embryos, and PGCs did not align with the SGPs. Consequently, several PGCs were seen scattered in the posterior. Eventually, these PGCs were unable to establish and/or maintain sustained contacts with the SGPs and were detected several diameters away from the coalesced gonads. Fifty-six percent of *dlg5* embryos showed five or more scattered PGCs (n = 52) as opposed to 17% of *balancer* embryos (n = 30). We also estimated the average number of germ cells lost per embryo in an independent experiment wherein we focused on total number of surviving PGCs in control as well as *dlg5* embryos. The embryos between stages 13 and 16 were used for the estimation. Consistently, we found that in the *dlg5* embryos, on average 4.4 PGCs per mutant embryo were lost (n = 18), as opposed to 1.7 PGCs in the *balancer* embryos (n = 20).

Loss of PGCs or scattering during gonad coalescence could arise due to multiple reasons, including misspecification or misalignment of SGPs, defective transmission, and/or reception of guidance cues. Moreover, aberrant migration could result because of germ cell autonomous defects or nonautonomous influence emanating from the surrounding somatic tissue. To assess whether *dlg-5* affects SGP specification, we stained these embryos with antibodies against a SGP marker, Eyes absent. Preliminary analysis suggests that total number of Eya-positive cells in dlg5 embryos are comparable with that detected in wild-type embryos, indicating that SGP specification is likely not affected in *dlg5* embryos. Although this observation points to a specific requirement of dlg5 during gonad coalescence, detailed analysis will be necessary to address the precise cause underlying loss of PGCs and whether dlg5 function is needed in the somatic component of the gonad, *i.e.*, SGPs or PGCs or in both cell types.

### Loss of Dlg5 leads to defects in E-cadherin distribution

**Figure 6:**
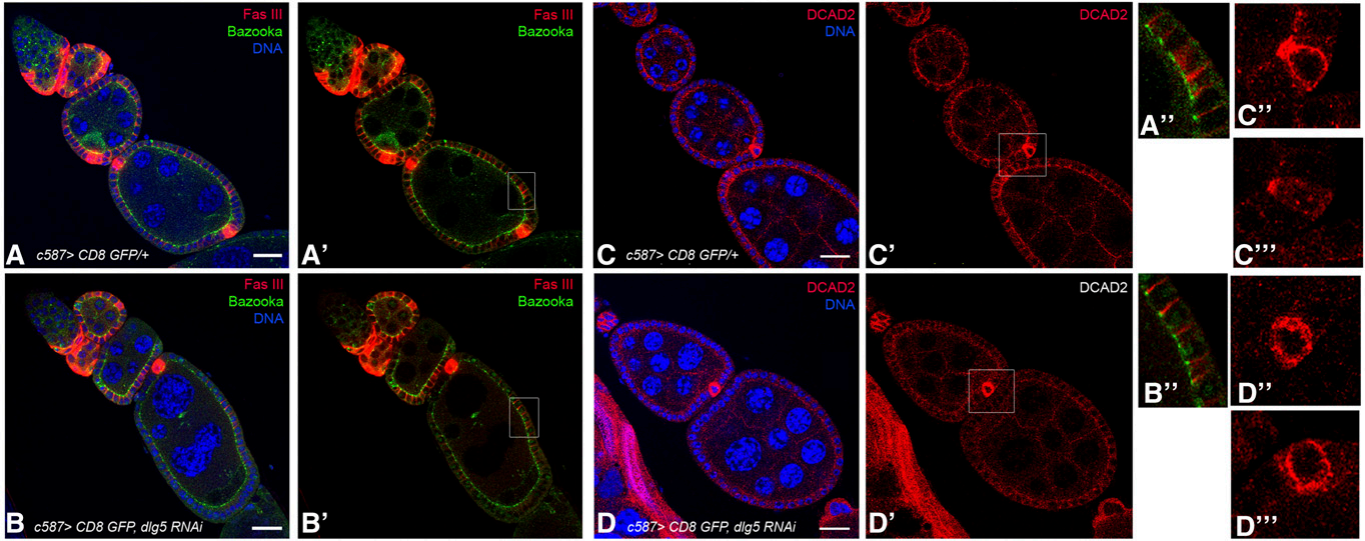
E-cadherin, but not Fasciclin III or Bazooka, is mislocalized in Dlg5 KD follicle cells. (A, A′) Control ovariole, driver only, and (B, B′) Dlg5 KD ovariole both show normal distribution of FasIII and Bazooka (higher magnification in A′′ and B′′). (C, C′) E-cad is more tightly localized in control ovarioles compared to its distribution in Dlg5 KD ovarioles. E-cad is asymmetrically localized on the apical side in control polar cells, but it looks uniformly distributed in Dlg5 KD polar cells (C′, C′′ and D′, D′′ show higher-resolution images of two examples). DNA, blue, scale bar, 20 μm.

To determine if the polarization of follicle cells is generally disrupted, we investigated the distribution of three proteins that are localized to specific membrane domains. The distribution of FasIII in the lateral membrane of Dlg5 KD cells was indistinguishable from wild-type (Figure 6, A, A′, B, and B′) (Nystul and Spradling 2010). The concentration of Bazooka (Baz) on the apical side of follicle cells was also not affected in Dlg5 KD cells (Figure 6, A, A′, A′′, B, B′, and B′′) (Abdelilah-Seyfried *et al.* 2003). It was therefore noteworthy that the distribution of DE-cadherin, the *Drosophila* homolog of E-cadherin, was less organized in Dlg5 KD follicle cells than in wild-type, giving an impression of lower levels of protein. Further, although E-cad showed high levels of protein in wild-type and mutant polar cells, the asymmetric accumulation of the protein was disrupted in Dlg5 KD polar cells (Figure 6 C, C′, C′′, D, D′, D′′).

The disruption of E-cadherin accumulation may contribute to the disorganized appearance of Dlg5 depleted ovaries. E-cadherin is typically upregulated in posterior follicle cells, and the oocyte preferentially associates with follicle cells expressing higher levels of DE-cadherin as the developing cyst enters region 3 (Godt and Tepass 1998; Gonzalez-Reyes and St. Johnston 1998). Oocyte mislocalization is frequently observed in females that have depleted Dlg5 levels in follicle cells (Figure 3C, C′). In some cases, the oocyte fails to associate at all with surrounding follicle cells and instead is located in the center of the nurse cells (Figure 4B′, arrow). Instances have also been observed in which the oocyte is completely mislocalized within the follicle (*i.e.*, a single oocyte is found at the anterior termini of the follicle), yet remains associated with follicle cells (not shown).

## Discussion

We found that *dlg5* is essential for normal division or maintenance of FSCs and GSCs, and is required in later stage follicle cells. Reduction of Dlg5 levels disrupts egg chamber formation, indicating that *dlg5* is involved in processes fundamental to egg chamber organization.

When Dlg5 was depleted in follicle cells for 4 d, epithelial follicle cells showed relatively normal cell shape and polarity, but the polar and stalk cells showed strong abnormalities in number, localization, and overall organization. The induction of ectopic polar cells and stalk cell overgrowth observed in these ovaries is significant because it has been suggested that the differentiation of these two cell types are controlled by similar signaling events, which distinguishes them from the other follicle cells (Tworoger *et al.* 1999; Zhang and Kalderon 2000). Loss of Dlg5 may interfere with the pathways that specify stalk and polar cell differentiation and maturation and, consequently, egg chamber organization.

The significantly enhanced phenotype observed upon depletion of Dlg5 in follicle cells for 10 d agrees with the *dlg5^D48^* clonal phenotype and is consistent with the proposition that the function of *dlg5* is completely lost in *dlg5^D48^*. Further, complete loss of *dlg5* function results in embryonic lethality, possibly also as a result of abnormal organization and integration of specific embryonic cells.

Our analysis of primitive embryonic gonad formation in *dlg5^D48^* embryos suggested that it likely functions during germ cell migration and gonad coalescence. These data are reminiscent of requirements of Dlg5 in the maintenance of the cohesion of migrating border cells in the ovary (Aranjuez *et al.* 2012). In this regard, it is interesting to note that proper gonad coalescence has been shown to depend on cell adhesion molecule E-cadherin (Jenkins *et al.* 2013). E-cadherin is consistently upregulated during late stages of gonad formation. Because loss of *dlg5* results in altered distribution of E-cadherin in follicle cells, it is conceivable that aberrant E-cadherin levels and/or localization could also contribute to the embryonic gonad-specific phenotypes. In this context, it will be interesting to analyze further the precise requirement and cell-type specificity of Dlg5 function in controlling cell adhesion and migration.

Many of the *dlg5* phenotypes we have described here could result from aberrant E-cadherin distribution. For instance, oocyte mislocalization is often seen as a result of loss or aberrant homophilic adhesion mediated by E-cadherin between posterior follicle cells and the oocyte (Godt and Tepass 1998; Gonzalez-Reyes and St. Johnston 1998). Formation of interfollicular stalks is also dependent on dynamic E-cadherin accumulation: E-cadherin accumulates first at the apical boundary of prefollicular cells, followed by the establishment of lateral cell contacts to initiate and complete intercalation to form a single wide stalk (Besse *et al.* 2002). Additionally, E-cadherin has been demonstrated to be required for recruiting and anchoring stem cells to their niche prior to adulthood in the *Drosophila* ovary (Song and Xie 2002; Song *et al.* 2002). By clonal analysis, we showed that both germline and follicle *dlg5* ovary stem cells are unable to give rise to normal daughter cells, indicating that the gene is essential in these cells. This may suggest several possibilities. There may be a cell-autonomous requirement for *dlg5* in follicle cells; however, the loss of stem cells may also be an indication of the requirement for E-cadherin-mediated adhesion to the stem cell niche. Finally, the lack of cohesion in migrating germ cells in *dlg5^D48^* embryos is consistent with a perturbation in cell–cell adhesion, as described above. The observed phenotypes, therefore, and the role of Dlg5 in E-cadherin distribution may be related; however, more work is needed to determine the nature of the participation of Dlg5 in E-cadherin distribution before any further conclusions may be drawn.

Dlg5 is involved in vesicle trafficking in vertebrates and has been reported to colocalize with several Rab GTPases (Nechiporuk *et al.* 2007; Chang *et al.* 2010). The punctate distribution of Dlg5 in the *Drosophila* ovary is consistent with a similar association of the protein with endosomes. Therefore, it seems possible that Dlg5 is involved in endosomal trafficking. The abnormal distribution of E-cadherin, which is recycled through the endosome, observed in Dlg5 KD ovaries supports this hypothesis (Jafar-Nejad *et al.* 2005; Langevin *et al.* 2005). Further, reduction of Dlg5 in the mammalian epithelial cell line LLc-PK1 resulted in lower levels of E-cadherin, but it is not clear how Dlg5 controls E-cadherin levels (Sezaki *et al.* 2012).

Thus, Dlg5, like its human homolog and Dlg1, may be involved in endosomal recycling of E-cad. But based on this characterization of *dlg5* and its functional requirement, there are fundamental differences between these two genes. Although *dlg5* shows embryonic lethality and is essential in ovarian stem cells, *dlg1* larvae can survive for >10 d and show overgrowth phenotypes in larvae and follicle cells. Persistent *dlg1* germ line clones develop into eggs, whereas follicle cells lacking *dlg1* sometimes show tumor-like invasion into the interior of the egg chamber (Goode and Perrimon 1997), a phenotype we did not observe in Dlg5 KD. The task of figuring out what each of these Dlg proteins contributes to apical protein trafficking and cell survival should prove informative and stimulating.
